# Long-term Chinese calligraphic handwriting training has a positive effect on brain network efficiency

**DOI:** 10.1371/journal.pone.0210962

**Published:** 2019-01-25

**Authors:** Wen Chen, Yong He, Chuansheng Chen, Ming Zhu, Suyu Bi, Jin Liu, Mingrui Xia, Qixiang Lin, Yiwen Wang, Wenjing Wang

**Affiliations:** 1 Advanced Innovation Center for Future Education, Beijing Normal University, Beijing, China; 2 State Key Laboratory of Cognitive Neuroscience and Learning, Beijing Normal University, Beijing, China; 3 IDG/McGovern Institute for Brain Research, Beijing Normal University, Beijing, China; 4 College of Information Science and Technology, Beijing Normal University, Beijing, China; 5 Department of Psychological Science, University of California Irvine, Irvine, California, United States of America; 6 School of International Journalism and Communication, Beijing Foreign Studies University, Beijing, China; 7 School of Arts and Media, Beijing Normal University, Beijing, China; University of Zurich, SWITZERLAND

## Abstract

As a visual art form, Chinese calligraphic handwriting (CCH) has been found to correlate with certain brain activity and to induce functional connectivity reorganization of the brain. This study investigated the effect of long-term CCH training on brain functional plasticity as assessed with network measures. With the resting-state fMRI data from 31 participants with at least five years of CCH training and 40 controls, we constructed brain functional networks, examined group differences at both the whole brain and modular levels, and correlated the topological characteristics with calligraphy skills. We found that, compared to the control group, the CCH group showed shorter characteristic path lengths and higher local efficiency in certain brain areas in the frontal and parietal cortices, limbic system, basal ganglia, and thalamus. Moreover, these network measures in the cingulate cortex, caudate nucleus, and thalamus were associated with CCH performance (i.e., copying and creating skills). These results suggest that long-term CCH training has a positive effect on the topological characteristics of brain networks.

## 1. Introduction

Chinese calligraphic handwriting (CCH) is a 3000-year-old art form. To master CCH skills requires years of intensive practice that involves sensory perception, motor skills, as well as multiple cognitive and emotional elements [1, 2]. Following previous research that found both structural and functional brain plasticity in response to many types of intensive training such as musical training [3, 4], driving [5], and juggling [6, 7], we have examined brain plasticity related to CCH training. Our previous two studies found that CCH training strengthened the RSFC of brain areas involved in updating and inhibition [8] and decreased the volume of the posterior cingulate cortex (PCC) [9].

In addition to the traditional univariate neuroimaging methods such as voxel-based morphometry (VBM) and resting-state functional connectivity (RSFC) used in the studies mentioned above, researchers have recently paid attention to brain connectivity networks or modular organization. Brain network analysis can mathematically describe various topological parameters of the brain’s organization in terms of graphs or networks, including the small-worldness, modularity, and regional network parameters [10, 11]. Studies have proved that functionally connected resting-state brain networks are associated with the anatomical connectivity of the brain [12, 13].

**Fig 1:**
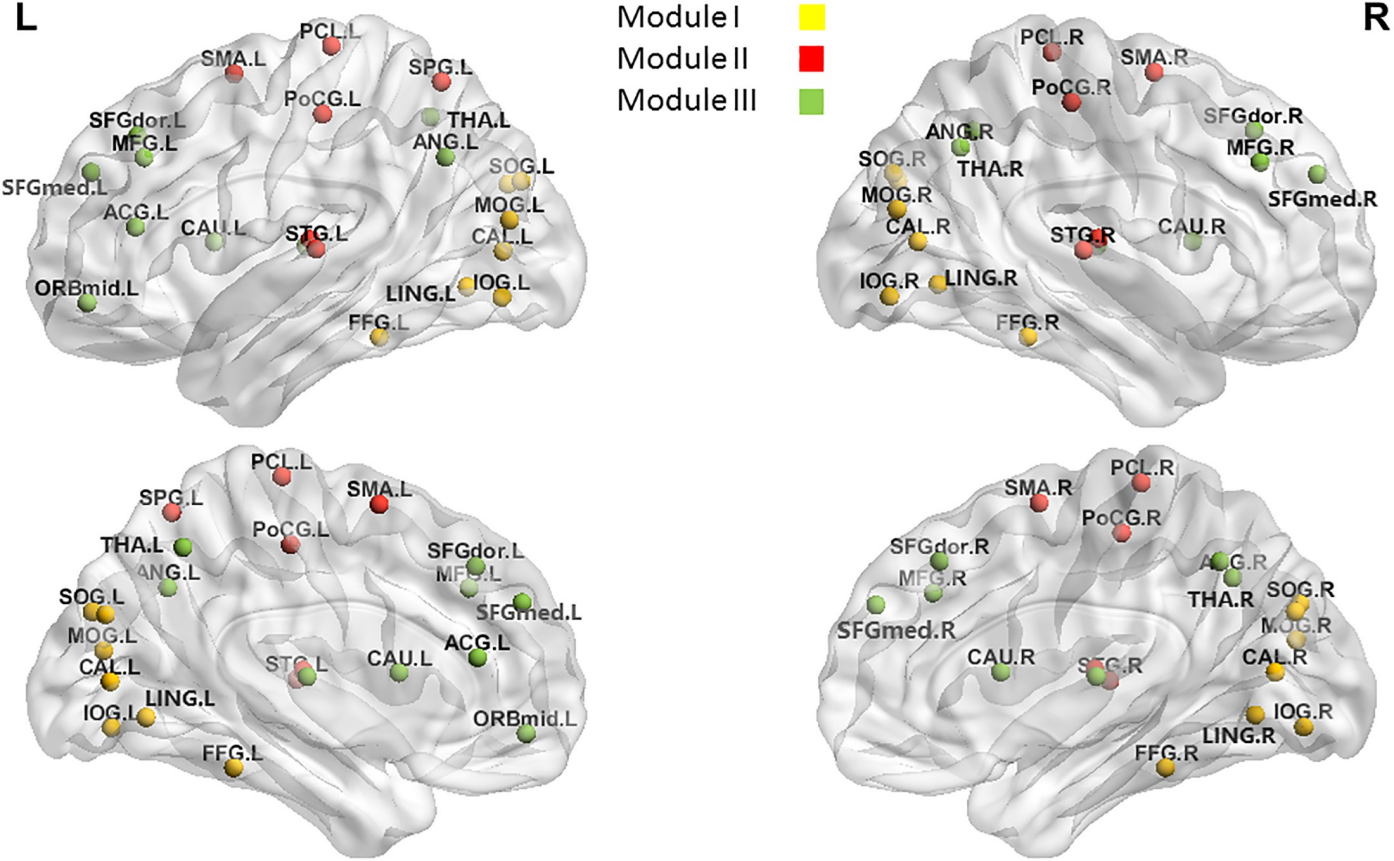
Visualization of the four modules selected for network efficiency analyses. Modules I, II, and III mean the sets of brain areas involved in visual processing, sensorimotor functions, and the DMN, respectively. L: left hemisphere; R: right hemisphere.

Given our previous findings of CCH training’s effects on the RSFC of certain brain areas [8], we hypothesized long-term CCH practicing would have an effect on the topological parameters of the resting-state brain network, including the frontal and parietal cortices, basal ganglia, and PCC. We explored the long-term CCH training’s effect on the topological characteristics of the whole brain and four specific modules. These modules were selected because of their relevance to visual processing (Module I), sensorimotor functions (Module II), and DMN (Module III), all of which are involved in CCH. More details of the brain areas included in each module are shown Fig 1 and S1 Table. Finally, within the CCH group, we further investigated the relationship between global and local network measures and calligraphy skills.

## 2. Materials and methods

### 2.1. Participants

Participants were recruited from Beijing Normal University, Beijing, China. The CCH group included 32 students who majored in calligraphy and had at least five years of formal training in CCH and the control group included 44 students who had no more than a few months of basic CCH skill training. All subjects were right-handed native Chinese speakers. Participants’ IQ was measured with Raven’s Advanced Progressive Matrices (APM) (for details, see Chen et al., 2017) [8]. Each participant signed an informed consent form after a full explanation of the study procedure. This study was approved by the Institutional Review Board of the State Key Laboratory of Cognitive Neuroscience and Learning at Beijing Normal University, China. Subjects were compensated for their time.

### 2.2 Calligraphy skills

Assessment of participants’ calligraphy skills was based on their performance on two tasks: copying a famous calligraphy work and creating a new calligraphy work.

All participants of the CCH group were asked to copy a part of a calligraphy masterpiece titled “the Northern Wei sculpture’ and to create a calligraphy work (in any style) using an existing quatrain written by Qi Gong, a celebrated cultural figure in China.

Participants’ works (with identity information removed) were evaluated by seven calligraphy teachers from the Department of Calligraphy of the School of Arts and Media, Beijing Normal University. They used a 10-point scoring system (from 1 to 10 with interval of 0.5). The final score for each piece of work was the mean of the five non-extreme scores after eliminating two extreme scores (the highest and the lowest) (see the Results section for interjudge reliability).

### 2.3 Brain imaging data collection and preprocessing

#### 2.3.1 fMRI data acquisition

All scanning was performed using a SIEMENS TRIO 3-Tesla scanner in the Brain Imaging Center of Beijing Normal University. Participants were told neither to have heavy physical activities nor have stimulating drinks the day before the scanning. Each participant underwent a 3D anatomic session and an eight-minute resting-state fMRI (RS-fMRI) scanning session. The 3D T1-weighted magnetization- prepared rapid gradient echo (MPRAGE) image was acquired with the following parameters: 144 sagital slices, slice thickness/gap = 1.3/0.65 mm, TR = 2530 ms, TE = 3.39 ms, inversion time (Ti) = 1100 ms, flip angle = 7°, FOV = 256×256 mm^2^, matrix size = 256×192. During the RS-fMRI session, the participants were instructed to keep their eyes closed, be as still as possible, and not to think about anything in particular. Images were obtained with the following parameters: 33 axial slices, thickness/gap = 3.5/0.7 mm, matrix size = 64×64, repetition time (TR) = 2000 ms, echo time (TE) = 30 ms, flip angle = 90°, field of view (FOV) = 200×200 mm^2^.

#### 2.3.2 Image preprocessing and analysis

Resting-State fMRI data were preprocessed using DPARSE (DPARSF, http://rfmri.org/DPARSF) [14]. Briefly, after discarding the first 10 volumes, the following steps were performed: correction, coregistration, segmentation, normalization, smoothing, linear detrending, regressing nuisance signals, and filtering. For more detail, see Chen et al. (2017).

In order to construct the connectivity network, the preprocessed RS-fMRI images data were overlapped with ALL templates (90 cortical and 26 cerebellar anatomical areas), and then the mean time series of all the 116 nodes were calculated in REST [15]. Pearson correlation was performed to obtain ‘r-value matrix’ and then Fisher’s r-to-z transformation was employed to obtain ‘z-score matrix’.

### 2.4 Statistics

After obtaining the correlation matrix, we used GraphVar (http://rfmri.org/GraphVar), a GUI-based toolbox for graph theoretical methods [16], to calculate the global and local clustering coefficient (*C*_p_), global and local characteristic path length (*L*_p_), global efficiency (*E*_glob_), and local efficiency (*E*_loc_) of the whole gray matter brain (90 ALL brain areas) and four modules with a range of network cost (0.1~0.5). We also generated 100 binary random networks per subject per threshold (null-model networks) to test the difference between network measures and the random networks, and also tested the small-worldness trait of human brain resting-state network. With age, gender and IQ as covariates, we then performed group analyses with 1000 permutations (corrected p <.05), which is a non-parametric testing method to detect the statistical significance of the group differences in network topological characteristics [16, 17]. The analyses were conducted both for the whole brain and the four modules.

Within the CCH group, we also correlated calligraphy skills (copying scores and creating scores) with topological characteristics (global and local *C*_p_, *L*_p_, *E*_glob_ and *E*_loc_).

## 3. Results

### 3.1 Reliability analysis of the calligraphy skills

We used Kendall’s coefficient of concordance to assess the agreement among the seven judges of the calligraphy skills. The Kendall’s W value was 0.719 (p< 0.001) for copying skills and 0.800 (p< 0.001) for creating skills. Because four of the seven judges were the participants’ teachers, we correlated the average ratings of the four calligraphy teachers of the participants and those of the three judges who had not taught these participants. Results showed very high correlations between them for the two measures of quality of CCH: the coping scores (r =.882, p <.01) and creating scores (r =.863, p <.01). These results suggest that even if the teachers might have been able to infer the identities of the participants from the anonymized writings, they were making similar judgments as the blind judges.

### 3.2 Demographic data

There was no group difference between the CCH and control groups in terms of gender, age (CCH group: 21.23±2.11; control group: 21.61±2.54), years of education (CCH group: 14.34±2.04; control group: 14.59±1.85), and IQ (CCH group: 26.47±3.68; control group: 27.58±3.74). The CCH practitioners had an average of 10 years of experience and started practicing at around 9 years of age (for more details, see Chen et. al, (2017).

Copying score was correlated negatively with the onset age of CCH practice (*r* = -0.627, *p*<0.001) and positively with the number of years of practice (*r* = 0.390, *p* = 0.021). Creating skills were significantly correlated with only the onset age of practice (*r* = -0.347, *p* = 0.041) (Table 1).

**Table 1:** The relationship between the time factors of CCH practice and calligraphy skills.

	Copying score		Creating score	
	*r*	*p*	*r*	*p*
**Onset age of practice**	-0.627	**0.000**	-0.347	**0.041**
**Yeas of practice**	0.390	**0.021**	0.164	0.346

### 3.3 Topological characteristics of the RSFC networks

#### 3.3.1 Whole brain network

Six topological properties, the global *C*_p_ and *L*_p_, local *C*_p_ and *L*_p_, *E*_glob_ and *E*_loc_, were used to characterize the global and local topological properties of the brain network. The two groups did not differ in small-world characteristics of brain organization (S1 Fig). They also did not differ in global *C*_p_, *L*_p_ and *E*_glob_. For the local parameters, we found that compared to the control group, the CCH group showed larger local *C*_p_ in right middle frontal gyrus, left inferior triangle frontal gyrus, left anterior and median cingulum, right hippocampus gyrus, right inferior frontal gyrus, right anterior cingulum, right calcarine fissure, and right fusiform gyrus (Table 2). The CCH group also showed shorter local *L*_p_ in right anteriror cingulum, left supramarginal gyrus, left caudate nucleus, and bilateral thalamus, but longer *L*_p_ in right hippocampus and caudate nucleus (Table 3). Finally, the CCH group showed higher *E*_loc_ in right PCC, right superior and inferior parietal gyrus, caudate nucleus, and thalamus (Table 4).

**Table 2:** Brain regions with significant group differences in characteristic path length between the CCH and control groups (corrected p <.05).

Series	AAL Template	Mean t value	Mean p value
32	Right anterior cingulate cortex	-0.387	0.039
38	Right hippocampus	0.665	0.037
63	Left supramarginal gyrus	-1.990	0.031
71	Left caudate nucleus	-1.815	0.013
72	Right caudate nucleus	0.300	0.010
77	Left thalamus	-0.706	0.025
78	Right thalamus	-1.615	0.024

Note: Positive t values mean that the CCH group had longer paths, whereas negative t values mean that the control group had longer paths.

**Table 3:** Brain regions with significant group differences in local clustering coefficient between the CCH and control groups (corrected p <.05).

Series	AAL Temple	Mean t value	Mean p value
8	Right middle frontal gyrus	0.267	0.033
14	Right inferior frontal gyrus	-0.778	0.043
15	Left inferior triangel frontal gyrus	1.166	0.045
31	Left anterior cingulate cortex	1.372	0.035
32	Right anterior cingulate cortex	-1.086	0.026
33	Left median cingulate cortex	0.057	0.040
34	Right median cingulate cortex	1.612	0.011
38	Right hippocampus gyrus	2.028	0.034
44	Right calcarine fissure	-0.368	0.039
56	Right fusiform gyrus	-0.679	0.007

Note: Positive t values mean that the CCH group had larger coefficients, whereas negative t values mean that the control group had larger coefficients.

**Table 4:** Brain regions with significant differences in local efficiency between the CCH and control groups (corrected p <.05).

Series	AAL Template	Mean t value	Mean p value
36	Right post cingulate cortex	0.292	0.019
60	Right superior parietal gyrus	0.317	0.034
62	Right inferior parietal gyrus	0.184	0.042
63	Left supramarginal gyrus	-0.465	0.023
72	Right caudate nucleus	0.683	0.027
78	Right thalamus	0.978	0.018

Note: Positive t values mean that the CCH group had higher local efficiency, whereas negative t values mean that the control group had higher local efficiency.

#### 3.3.2 Group difference in modular network efficiency

**Fig 2:**
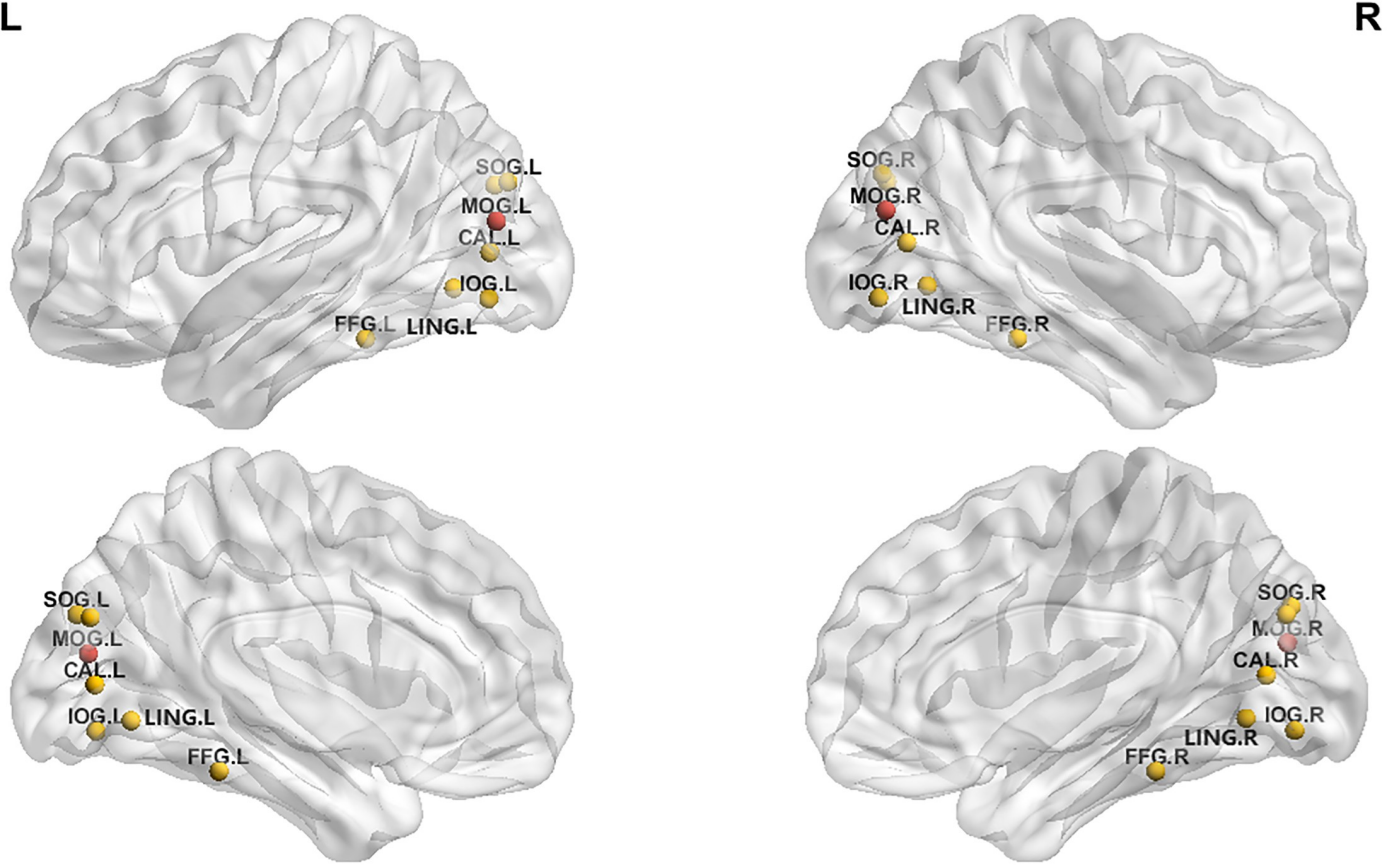
Bilateral middle occipital region showed higher *E*_loc_ in the CCH group than in the control group (red) in Module I, with 1000 permutations (p <.05). Note: 1. Calcarine_L; 2. Calcarine_R; 3. Cuneus_L; 4. Cuneus_R; 5. Lingual_L; 6. Lingual_R; 7. Occipital_Sup_L; 8. Occipital_Sup_R; 9. Occipital_Mid_L; 10. Occipital_Mid_R; 11. Occipital_Inf_L; 12. Occipital_Inf_R; 13. Fusiform_L; 14. Fusiform_R.

We chose four modular networks that are most relevant to CCH training for the network efficiency analysis. Module I (visual processing) showed that the CCH group had high lower *C*_p_ in right calcarine fissure and higher *C*_p_ in left MOG than did the control group (corrected alpha level of 0.05 with 1000 permutations, at the threshold of 0.19–0.45). The CCH group also showed longer *L*_p_ in right inferior occipital gyrus and shorter *L*_p_ in left fusiform. The CCH group showed higher *E*_loc_ in bilateral middle occipital gyrus (Fig 2). Finally, no group difference was found in terms of *E*_global_ in Module I.

**Fig 3:**
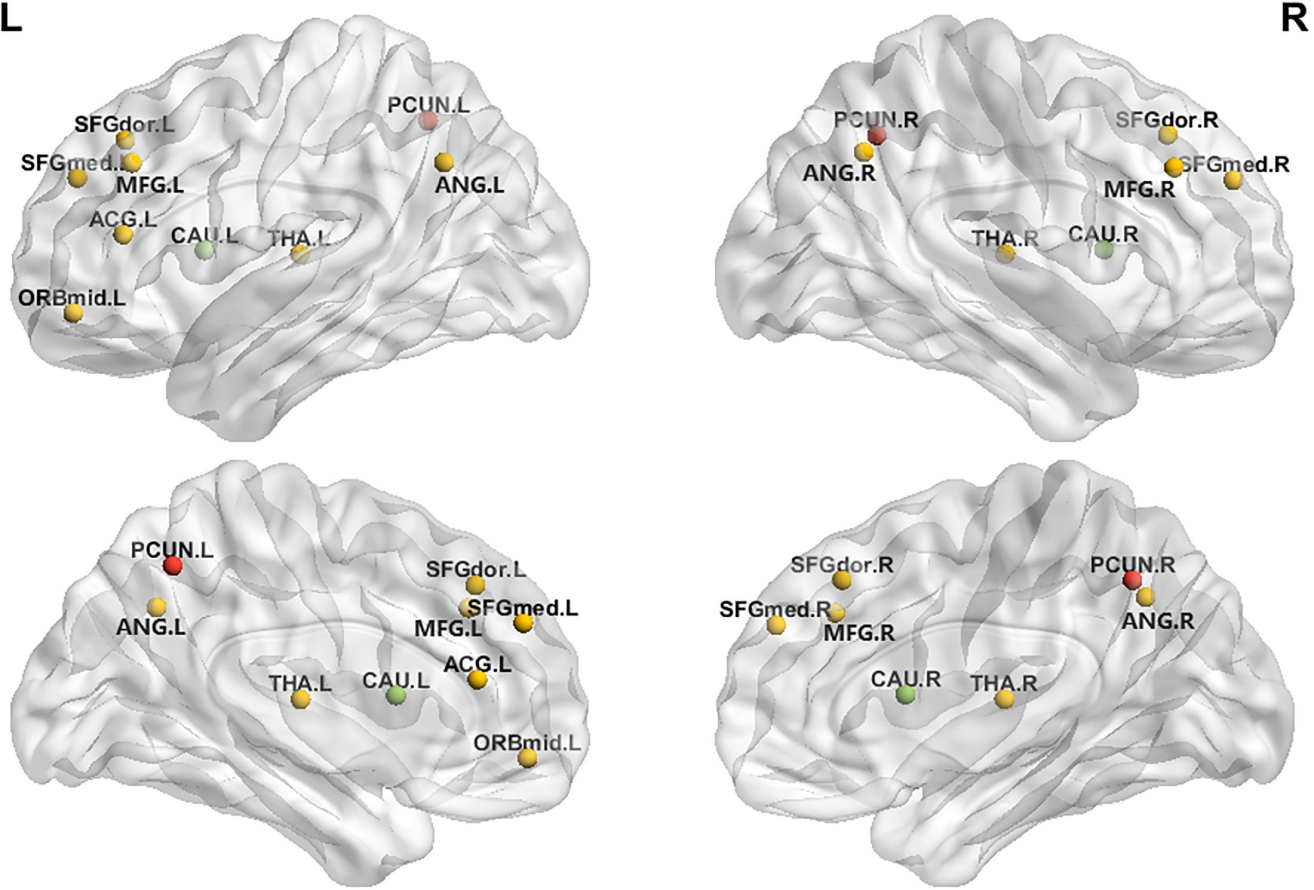
The CCH group showed higher *E*_loc_ in bilateral precuneus (red) and lower in bilateral caudate nucleus (green) than the control group in Module III. Note: 1. Frontal_Sup_L (dorsolateral); 2. Frontal_Sup_R (dorsolateral); 3. Frontal_Mid_L; 4. Frontal_Mid_R; 5. Frontal_Mid_Orb_L; 6. Frontal_Sup_Medial_L; 7. Frontal_Sup_Medial_R; 8. Cingulum_Ant_L (Anterior cingulate and paracingulate gyri); 9. Angular_L; 10. Angular_R; 11. Precuneus_L; 12. Precuneus_R; 13. Caudate_L; 14. Caudate_R; 15. Thalamus_L; 16.Thalamus_R.

Contrary to our expectations, Module II (the sensorimotor areas) showed no significant group differences. For Module III (the DMN), the CCH group showed higher *C*_p_ in left superior and medial frontal gyrus and lower *C*_p_ in bilateral precuneus than did the control group. The CCH group also showed significantly shorter *L*_p_ when the network threshold was 0.15, 0.16, 0.19 and 0.20. At the local level, the CCH group showed significantly shorter *L*_p_ than the control group in left anterior cingulum and bilateral precuneus, but higher *L*_p_ in right caudate nucleus. In contrast, the CCH group showed significantly higher *E*_loc_ in bilateral precuneus, and lower *E*_loc_ in bilateral caudate nucleus (Fig 3). The CCH group tended to have higher *E*_global_ than the control group, which reached significance at two network thresholds (0.16 and 0.20) (S2 Fig).

### 3.4 Correlation analyses between brain network parameters and calligraphy skills

The above results showed group differences in topological characteristics (shorter *L*_p_ and higher *E*_loc_ for the CCH group than the control group). To extend those results to individual differences within the CCH group, we conducted correlation analyses between brain network parameters and calligraphy skills. The correlational analyses generally confirmed those associations within the CCH group. Specifically, within thresholds 0.13~0.21, copying score was negatively correlated with characteristic path length at the global level. At the local level, the *L*_p_ of certain brain regions (i.e., bilateral olfactory cortex, amygdala, caudate nucleus; right rectus, thalamus and middle temporal pole) all showed negative relationship with copying score, whereas the *L*_p_ of left thalamus was negatively correlated with creating score and the *L*_p_ of left inferior occipital gyrus and bilateral caudate nucleus was positively correlated with creating score.

*C*_p_ in the right supplementary motor areas, right superior occipital gyrus, and left inferior occipital gyrus was negatively correlated with copying scores, but *C*_p_ in right gyrus rectus and amygdala was positively correlated with copying score. *C*_p_ in the right calcarine fissure, cuneus, superior and middle occipital gyrus, middle temporal gyrus, and left inferior occipital gyrus was negatively correlated with creating score, and that of right inferior frontal gyrus was positively correlated with creating score.

The *E*_loc_ of bilateral superior medial frontal gyrus and PCC, right hippocampus and lenticular nucleus (pallidum), and left temporal pole (middle temporal gyrus) showed positive relationship with copying score. The *E*_loc_ of left supplementary motor area, insula, bilateral paracentral lobule, caudate nucleus and thalamus, and right lenticular nucleus (pallidum) was positively correlated with creating score, but *E*_loc_ of left inferior occipital gyrus was negatively correlated with creating score (Table 5).

**Table 5:** The positive (+) and negative (-) correlation brain regions with r values between local topological characteristics (i. e., *L*_p_, *C*_p_, *E*_loc_) and calligraphy skills.

	Copying score		Creating score	
	+	-	+	-
***L***_**p**_	N	left olfactory cortex (-0.374)	left inferior occipital gyrus (0.567)	left thalamus (-0.249)
		right olfactory cortex (-0.362)	left caudata nucelus (0.170)	
		left amygdala (-0.453)	right caudata nucelus (0.150)	
		right amygdala (-0.558)		
		left caudate nucelus (-0.317)		
		right caudate nucelus (-0.268)		
		right rectus (-0.439)		
		right thalamus (-0.346)		
		right middle temporal pole (-0.623)		
***C***_**p**_	right gyrus rectus (0.378)	right supplementary motor areas (-0.566)	right inferior frontal gyrus (0.524)	right calcarine fissure (-0.356)
	right amygdala (0.438)	right superior occipital gyrus (-0.482)		right cuneus (-0.359)
		left inferior occipital gyrus (-0.456)		right superior occipital gyrus (-0.395)
				right middle occipital gyrus (-0.357)
				right middle temporal gyrus (-0.345)
				left inferior occipital gyrus (-0.386)
***E***_**loc**_	left superior medial frontal gyrus (0.604)	right paracentral lobule (-0.355)	left supplementary motor area (0.422)	left inferior occipital gyrus (-0.440)
	right superior medial frontal gyrus (0.671)	right caudate nucelus (-0.118)	left insula (0.439)	
	left post cingulum cortex (0.567)		left paracentral lobule (0.366)	
	right post cingulum cortex (0.657)		right paracentral lobule (0.257)	
	right hippocampus (0.442)		left caudata nucelus (0.156)	
	right lenticular nucleus (pallidum) (0.534)		right caudata nucelus (0.182)	
	left temporal pole (middle temporal gyrus) (0.419)		left thalamus (0.164)	
			right thalamus (0.188)	
			right lenticular nucleus (pallidum) (0.387)	

Note: + positive correlation (P < 0.05),

− negative correlation (P < 0.05),

N no significant correlation brain regions (P > 0.05).

Taken together, we found that the cingulate cortex, caudate nucleus and thalamus were the core brain areas that showed both group differences between the CCH and control groups and significant correlations with calligraphy skills within the CCH group (Table 6).

**Table 6:** Group differences and neural correlates (within the DMN) of CCH skills.

Brain areas	Group difference		Correlation			
			Coping score		Creating score	
	*L*_p_	*E*_loc_	*L*_p_	*E*_loc_	*L*_p_	*E*_loc_
Left anterior cingulate cortex						
Right anterior cingulate cortex	-					
Left post cingulate cortex				+		
Right post cingulate cortex		+		+		
Left caudate nucleus	-		-		+	+
Right caudate nucleus	+	+	-	-	+	+
Left thalamus	-				-	+
Right thalamus	-	+	-			+

+ means the CCH group had longer L_p_/higher *E*_loc_ than the control group or positive correlation.

− means the CCH group had shorter *L*_p_/lower *E*_loc_ than the control group or negative correlation (P < 0.05).

## 4. Discussion

The current study explored the effect the long-term experience with CCH on brain network efficiency assessed with parameters based on graph theory. We found that compared to the controls, individuals with long-term CCH training showed advantages in topological characteristics (i.e., *L*_p_, *C*_p_ and *E*_loc_) in certain brain areas based on both whole brain and modular analyses. Moreover, within the CCH group, calligraphy skills were associated with brain network efficiency parameters, especially *L*_p_ and *E*_loc_.

Seven brain regions showed significant group differences in *L*_p_ (Table 3), with the CCH group having shorter *L*_p_ than the control group for five of the seven regions. It appears that CCH training increased the information transfer speed (as indexed by *L*_p_ from one brain area to another). Consistent with the group differences, copying score had a significant negative relationship with *L*_p_ within CCH participants. Similar to the results with *L*_p_, the CCH group had higher *E*_loc_ than the control group in all brain areas in the right hemisphere and *E*_loc_ in many of these brain regions was positively correlated with calligraphy skills (including both copying and creating scores) of CCH participants. Those results demonstrated that CCH training improved the partial information network’s topological structure of certain brain areas.

These results add to the literature on the significant role of structural and functional brain network efficiency in behavior. For example, increased *L*_p_ and/or decreased *E*_loc_ are often associated with aging [18] and various kinds of brain diseases [19, 20]. Decreased efficiency is often associated with a disrupted network related to brain disease [21, 22].On the other hand, higher efficiency and shorter path length have been linked to a higher intelligence quotient (IQ) [23, 24] in diffusion tensor imaging tractography and RSFC studies [25]. Our study showed that long-term CCH training had positive effects on topological characteristics of the resting-state brain network.

Our finding that the brain regions being affected by CCH training are in the frontal and parietal gyri, limbic system, basal ganglia, and thalamus is consistent our previous analysis with different methods (Chen, et al., 2017). Other forms of art training also seem to share some common effects. For example, CCH and painting training (another form of visual art) would both impact brain areas associated with the executive attention, cognitive control, and motor planning [26]. Musical improvisation has also been correlated with the DMN [27] and long-term musical training with stronger brain functional connectivity between the anterior cingulate cortex, right angular gyrus, and bilateral superior frontal gyrus [28]. However, music training also affects an extensive brain network related to auditory, cognitive, motor, and emotional processing [29], which is different from the CCH-related brain areas. Finally, dancing and piano training has been associated with areas different from those for CCH practice. For example, dancing training is associated with bilateral cerebellum and piano training with the parietal cortex and bilateral cerebellum [30].

As a part of the limbic system, the cingulate cortex plays an important role in emotional processes [31–33]. One recent study found that treatment for anxiety led to higher activation in the cingulate cortex and that the extent of reduction in anxiety was positively correlated with increases in activation [34]. Another study found that the strength of intrinsic connectivity between the PCC and the dorsal attention network was positively correlated with clinical improvements among patients suffering from chronic pain [35]. Emotional processes might have accounted for the effect of CCH training on *E*_loc_ of the cingulate cortex because CCH may lower the level of anxiety and lead to stable mood.

In terms of bilateral thalamus that showed shorter *L*_p_ and higher *E*_loc_ in the CCH group than the control group (as well as significant correlations with better calligraphy skills), it is likely due to the fact that the thalamus is the major center for sensory information processing, including relaying the motor signals. Previous studies have shown that the thalamus plays an important role in the early stages of new learning [36], inhibitory control [37], and motor control [38], all of which are integral to CCH training.

Unlike the cingulate cortex and thalamus, the caudate nucleus showed somewhat inconsistent results (depending on hemisphere, level of analysis [whole brain or modular], and group vs. individual differences). The caudate nucleus is associated with motor process and cognitive functions. For example, activation in the caudate nucleus was greater during spatial and motoric memory tasks than during a nonspatial task [39]. Other studies found that the caudate nucleus showed higher activities in perceptual-motor tasks than in control conditions [40, 41]. Because the hippocampus and the striatum (caudate nucleus and putamen) are two different memory systems involved in place/spatial learning [42, 43], we speculated that these parallel systems might have complicated the relationship between the brain network and calligraphy training.

Finally, the superior medial frontal cortex (SMFC) was correlated with better calligraphy skills. SMFC play a vital role in inhibitory control [44] and the prepotent motor response [45, 46]. Our results underlined the important role of the inhibitory system in the CCH training.

In the current study, we did not find group difference in Module III, which is associated with sensory and motor functions. This result suggests that CCH training does not require more network efficiency than the regular (non-CCH) writing that has to be done by any students. Instead, CCH training seems to affect brain areas for higher cognitive abilities, such as inhibition.

It is also worth mentioning that this study involved quantitative ratings of calligraphy skills, which surprisingly has not been attempted in previous studies of calligraphy. We found that calligraphy skills were, as would be expected, negatively associated with the onset age of CCH practice. It is not clear though whether CCH may have a sensitive period as other kinds of skill acquisition [47, 48].We found that the number of years of CCH practice was correlated with the copying score, but not with the creating score, which is consistent with the common phenomenon that it is relatively easy to learn CCH but quite difficult to reach a mastery level with creative products. We correlated ‘Onset age of practice’ and ‘Years of practice’ with the brain data, but no brain areas survived the multiple correction. This result suggested that CCH level was a better index than were the onset age of practice or years of practice, even though the latter two were also correlated with performance. In other words, it was not how early one started training or how long one was trained, but how well one was trained that made a difference in neural correlates. Finally, although both copying and creating scores were correlated with the DMN, which plays a vital role in the creative process (including artistic creations) [49–52], there were subtle differences in the neural correlates of the two aspects of CCH performance. Whereas copying scores were associated with the *L*_P_ and *E*_loc_ within a relatively widespread network of brain areas, creating scores were correlated with only a few brain areas, mostly the thalamus. Consistent with our results, the thalamus has been linked to creativity [53].

In sum, this study found that long-term CCH training had a positive effect on the efficiency of the resting-state brain network, with visual and DMN-related brain areas showing shorter *L*_p_ and higher *E*_loc_ for CCH participants than for the controls and with those brain parameters being correlated with better calligraphy skills of CCH participants. However, with a cross-sectional study, the results we found in the current study could not rule out alternative explanatory variables such as training in other art forms (e.g. painting) or personality correlates, nor could we examine potential mediators such as personality traits. Future studies should use a longitudinal design or a randomized training design to examine the causal relations and potential mechanisms.

## Supporting information

S1 Fig Both the CCH and control groups revealed small-world characteristics in their resting-state brain network. σ = (Cnet/Crand)/(lnet/lrand),σ>1 means the network owns the smallworldness.The two groups showed virtually the same results (overlapping lines), and hence the group x smallworldness interaction was not significant (indicated by empty circles) for any threshold.
(TIF)

S2 Fig The global efficiency of the DMN. It was significantly higher for the CCH group than the control group at two network thresholds,.16 and.20 (as indicated by the filled circles for the group x efficiency interaction.
(TIF)

S1 Table Brain areas (from the AAL template) of the four modules. (DOCX)
