# Supplementary figures and images for: Long-term Chinese calligraphic handwriting training has a positive effect on brain network efficiency
Source: PLoS One. 2019 Jan 25;14(1):e0210962. doi: 10.1371/journal.pone.0210962 (PMC6347361; doi:10.1371/journal.pone.0210962)

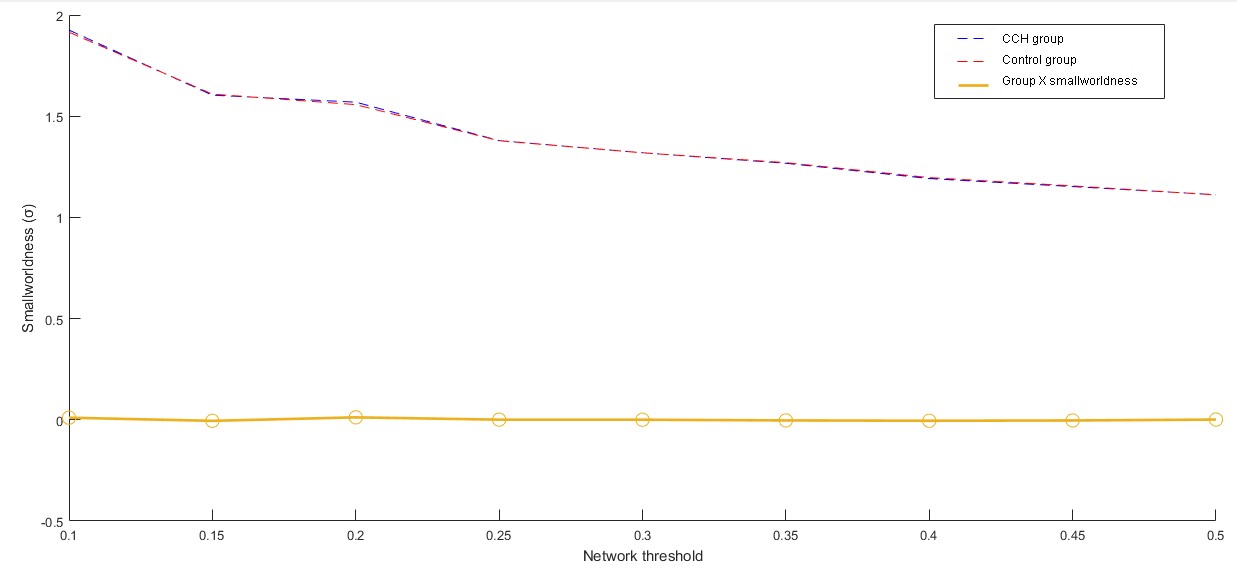

Supplement: S1 Fig — σ = (Cnet/Crand)/(lnet/lrand),σ>1 means the network owns the smallworldness.The two groups showed virtually the same results (overlapping lines), and hence the group x smallworldness interaction was not significant (indicated by empty circles) for any threshold. (TIF) [file pone.0210962.s001.tif]

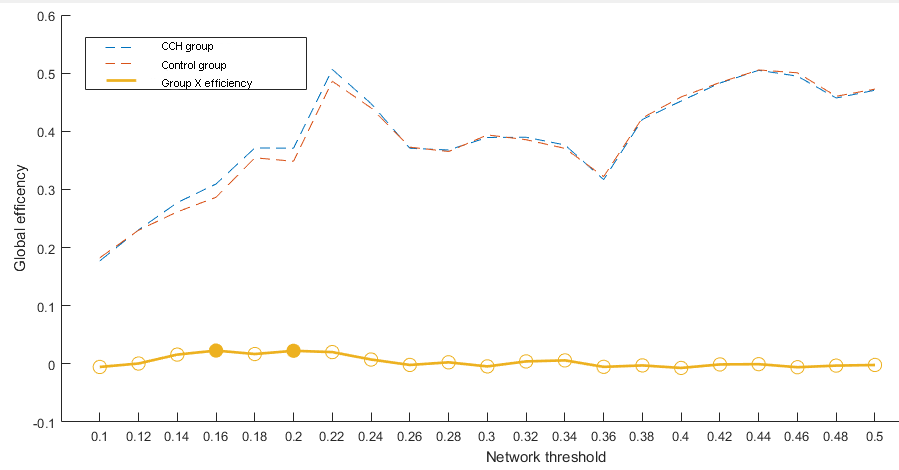

Supplement: S2 Fig — It was significantly higher for the CCH group than the control group at two network thresholds,.16 and.20 (as indicated by the filled circles for the group x efficiency interaction. (TIF) [file pone.0210962.s002.tif]
