# Supplementary material for: Long-term Chinese calligraphic handwriting training has a positive effect on brain network efficiency
Source: PLoS One. 2019 Jan 25;14(1):e0210962. doi: 10.1371/journal.pone.0210962 (PMC6347361; doi:10.1371/journal.pone.0210962)
Supplement: S1 Table — (DOCX) [file pone.0210962.s003.docx]

Long-term Chinese calligraphic handwriting training has a positive effect on brain network efficiency

*Supporting Information*

**S1 Table. Brain areas (from the AAL template) of the four modules.**

| **Modules** | **Networks** | **Number of Brain Areas** | **AAL_template** | **Abbreviations** |
| --- | --- | --- | --- | --- |
| I | Visual Network | 1 | Calcarine_L | CAL.L |
|  |  | 2 | Calcarine_R | CAL.R |
|  |  | 3 | Cuneus_L | CUN.L |
|  |  | 4 | Cuneus_R | CUN.R |
|  |  | 5 | Lingual_L | LING.L |
|  |  | 6 | Lingual_R | LING.R |
|  |  | 7 | Occipital_Sup_L | SOG.L |
|  |  | 8 | Occipital_Sup_R | SOG.R |
|  |  | 9 | Occipital_Mid_L | MOG.L |
|  |  | 10 | Occipital_Mid_R | MOG.R |
|  |  | 11 | Occipital_Inf_L | IOG.L |
|  |  | 12 | Occipital_Inf_R | IOG.R |
|  |  | 13 | Fusiform_L | FFG.L |
|  |  | 14 | Fusiform_R | FFG.R |
| II | Sensorimotor Network | 1 | Supp_Motor_Area_L | SMA.L |
|  |  | 2 | Supp_Motor_Area_R | SMA.R |
|  |  | 3 | Postcentral_L | PoCG.L |
|  |  | 4 | Postcentral_R | PoCG.R |
|  |  | 5 | Parietal_Sup_L | SPG.L |
|  |  | 6 | Paracentral-Lobule_L | PCL.L |
|  |  | 7 | Paracentral_Lobule_R | PCL.R |
|  |  | 8 | Heschl_L | HES.L |
|  |  | 9 | Heschl_R | HES.R |
|  |  | 10 | Temporal_Sup_L | STG.L |
|  |  | 11 | Temporal_Sup_R | STG.R |
| III | Default Mode Network | 1 | Frontal_Sup_L(dorsolateral) | SFGdor.L |
|  |  | 2 | Frontal_Sup_R(dorsolateral) | SFGdor.R |
|  |  | 3 | Frontal_Mid_L | MFG.L |
|  |  | 4 | Frontal_Mid_R | MFG.R |
|  |  | 5 | Frontal_Mid_Orb_L | ORBmid.L |
|  |  | 6 | Frontal_Sup_Medial_L | SFGmed.L |
|  |  | 7 | Frontal_Sup_Medial_R | SFGmed.R |
|  |  | 8 | Cingulum_Ant_L(Anterior cingulate and paracingulate gyri) | ACG.L |
|  |  | 9 | Angular_L | ANG.L |
|  |  | 10 | Angular_R | ANG.R |
|  |  | 11 | Precuneus_L | PCUN.L |
|  |  | 12 | Precuneus_R | PCUN.R |
|  |  | 13 | Caudate_L | CAU.L |
|  |  | 14 | Caudate_R | CAU.R |
|  |  | 15 | Thalamus_L | THA.L |
|  |  | 16 | Thalamus_R | THA.R |
